# Comparison of immediate versus optional delayed surgical repair for treatment of acute anterior cruciate ligament injury through a parallel, multicentric, pragmatic randomized controlled trial – IODA trial

**DOI:** 10.1186/s13102-024-00816-6

**Published:** 2024-01-18

**Authors:** Annemie Smeets, Feryal Ghafelzadeh Ahwaz, Stijn Bogaerts, Pieter Berger, Koen Peers

**Affiliations:** 1 Research group of Physical & Rehabilitation Medicine, Department of Development & Regeneration, KU Leuven, Leuven, Belgium; 2 Research group of Musculoskeletal Rehabilitation, Department of Rehabilitation Sciences & Physiotherapy, KU Leuven, Leuven, Belgium; 3 Department of Physical & Rehabilitation Medicine, University Hospitals Leuven, Leuven, Belgium; 4 Department of Orthopaedic Surgery, University of Leuven, Leuven, Belgium

**Keywords:** Anterior cruciate ligament injury, RCT, Conservative therapy, ACL reconstruction

## Abstract

**Background:**

Standard care for anterior cruciate ligament (ACL) injuries often includes surgical reconstruction of the ACL. However, two randomized controlled trials [1, 2] (RCT) concluded that conservative treatment does not result in inferior clinical outcomes compared to immediate ACL reconstruction. More research is needed to verify these results and assess whether patient-specific parameters determine if a patient would benefit from one treatment option over the other.

**Methods:**

This is a pragmatic, multi-center RCT with two parallel groups. Patients with an acute ACL injury will be recruited from Belgian hospitals. Patients will be randomized to conservative treatment (rehabilitation + optional delayed surgery) or immediate ACL reconstruction (< 12 weeks). The primary outcome is the Knee injury and Osteoarthritis Outcome Score (KOOS) at 7 months (short term) and 1-year long term) post-injury. These following additional outcomes will be administered at 4 and 7 months (short term) and 1, 2, and 3 years post-injury (long term): patient-reported outcomes concerning knee symptoms, knee function and quality of life, functional knee tests, time to return to pre-injury activity level and return to work, structural knee joint damage and cartilage health (only at 4 months and 3 years post-injury), as well as adverse events such as re-rupture rates. Furthermore, the secondary objective is to identify (through a predictive analysis) individuals who would benefit the most from early reconstruction versus those who should rather be treated conservatively.

**Discussion:**

This large RCT will assess the clinical effectiveness of both surgical and conservative treatment. In addition, it will be the first study that provides insights into which patient-specific factors predict successful outcomes after conservative treatment of ACL injuries. These results will be the first step toward early patient identification regarding treatment decisions. This is urgently needed to avoid (1) delayed surgeries and prolonged rehabilitation and (2) unnecessary surgeries.

**Trial registration:**

this trial was registered on ClinicalTrials.gov (NCT05747079) on 10/02/2023.

**Supplementary Information:**

The online version contains supplementary material available at 10.1186/s13102-024-00816-6.

## Background

Rupture of the anterior cruciate ligament (ACL) is a common injury, especially in young, physically active individuals, with an indicate rate of 0.7/1000 person-years in Belgium [3]. The ACL has an important role in both static and dynamic stabilisation of the knee joint with a primary role limiting anterior translation of the tibia relative to the femur. In more than 70% of the injuries, the rupture occurs during a non-contact mechanism, such as a sudden change of direction or landing with the knee near full extension [4]. Frequently, not only the ACL is ruptured but typically also injuries to the menisci, cartilage, collateral ligaments and subchondral bone are present. The rupture of the ACL and damage to other knee stabilising structures, often results in knee joint instability affecting daily activities and sports leading to poor knee related quality of life. Furthermore, ACL injuries are associated with increased risk of post traumatic knee osteoarthritis (PTOA) [5] and athletes who return to sport have a high risk to sustain a re-injury [6]. The medical community has always been convinced that surgical repair of the ACL was necessary to restore mechanical knee stability so that patients can safely return to sports [7], but also to avoid long term disadvantages such as persistent knee instability, re-injury [6] and PTOA [5, 8]. However, evidence assessing treatment outcomes after ACL reconstruction does not support these beliefs. Ardern et al. [9] found that only 55% of athletes who underwent an ACL reconstruction return to their preinjury sport level. Furthermore, a recent meta-analysis showed that about 50% of the patients who underwent surgical repair of their ACL have cartilage degeneration 20 years after surgery [10] and up to 23% suffer a new ACL injury (ipsilateral or contralateral) within two years after return to sport [11]. Based on these results, one can assume that ACL reconstruction offers no certainty of restoration of normal knee function and protection from long-term disadvantages. Besides, it is uncertain whether ACL reconstruction results in benefits at all compared to conservative treatment. In total, 3 RCTs compared the clinical effectiveness between ACL reconstruction and conservative treatment (= rehabilitation + optional delayed surgery): the KANON trial [1] COMPARE trial [2] and ACL SNNAP [12]). The KANON trial concluded that a strategy of early reconstruction plus rehabilitation did not provide better results at five years than a strategy of initial rehabilitation with optional delayed ACL reconstruction [1, 13].The COMPARE trial found slightly better self-reported outcomes (knee symptoms, self-reported knee function, and perception of the ability to participate in sports) in the immediate ACL reconstruction group compared with the conservative group at two years follow-up. However, none of these findings were considered clinically important. The SNAPP trial investigated chronic ACL ruptures and found that patients with chronic symptomatic ACL deficiency have better clinical outcomes if they undergo surgery [12].

Based on the results of the KANON trial [1] and the COMPARE trial [2] one can conclude that conservative management with optional delayed surgery does not result in inferior clinical outcomes compared to immediate ACL reconstruction on a population level [14].

Though, on the level of the individual patient, large between-subject differences were found. In the KANON trial, 39% of the ACL patients in the conservative treatment group showed persistent knee instability requiring delayed surgery during the two years follow up, this percentage has grown to 51% at the five years follow up [1, 13]. The compare trial reported that 50% of the ACL patients in the conservative group required delayed surgery in the two years follow up [2]. In this group of patients, time to return-to-sport is extended, and longer sick leave times are observed because surgery is delayed compared to patients undergoing immediate ACL reconstruction [15]. Hence, early identification of patients who would benefit from early ACL reconstruction, or on the contrary, from rehabilitation alone, is crucial to reduce resource consumption and decrease irrelevant overtreatment. It is hypothesized that treatment success relies on clinical factors (such as knee function and MRI features [16] ) as well as the quality of rehabilitation [17], and psychological factors such as expectations [18], fear of re-injury [19, 20] and locus of control [21].

This large RCT has 2 aims: (1) to compare the clinical effectiveness of both treatment options and as such verify the existing literature, and (2) to assesses which patient-specific factors predict successful outcomes after conservative treatment of ACL injuries.

## Objectives

### Primary objective

The primary objective of this study is to compare the clinical effectiveness between immediate ACL reconstruction and conservative treatment with optional delayed surgical reconstruction of acute ACL injuries. Outcome parameters of interest are knee outcomes reported by the patients themselves. The primary outcome is the KOOS QOL.

This primary objective is translated in the following hypotheses:

- The optional delayed surgery approach for acute ACL injury will result in non-inferior patient reported outcome measures at long term, i.e. 1 year post-injury, compared to immediate ACL reconstruction.
- The optional delayed surgery approach for acute ACL injury will result in better patient reported outcome measures at short term, i.e. 7 months post-injury, compared to immediate ACL reconstruction.

### Secondary objective

The secondary objective is to identify individuals who would benefit the most from reconstruction versus those who should rather be treated conservatively. A prediction model will be built in the intervention group (rehabilitation with optional delayed ACL reconstruction) to investigate this. Five predictor variables will be considered to predict whether delayed reconstruction is necessary. The following five variables will be considered:

1. KOOS QOL: patients with high scores on the KOOS QOL are less likely to require delayed ACL surgery [22].
2. MRI features: patients with pathologic MRI features (fiber continuity disturbed, high/heterogenous signal intensity, more horizontal slope of ACL, unclear boundaries) are more likely to require delayed ACL surgery [16].
3. hemarthrosis: patients with a lower degree of hemarthrosis detected by MRI are less likely to require delayed ACL surgery.
4. IPQ-R (illness perception questionnaire): patients’ expectations play a crucial role in determining the success of non-surgical treatment [23].
5. pre-activity level: patients who are less physically active prior to their injury are less likely to require delayed ACL surgery [24].

## Methods/design

### Study design

This is a pragmatic, multi-center, randomized controlled trial with two parallel groups: [1] conservative treatment (consisting of rehabilitation + optional delayed surgery) and [2] immediate ACL reconstruction in patients with an acute ACL injury.

The protocol is conform the Standard Protocol Items: Recommendations for Interventional Trials (SPIRIT) guidelines [25] (the SPIRIT checklist is provided as Additional file 1).

### Participants

#### Eligibility criteria

Participants eligible for inclusion in this trial must meet all of the following criteria:

1. Rotational trauma to a previously non-injured knee for which medical advice was sought within 4 weeks after injury.
2. Medical diagnosis of ACL insufficiency including MRI (both partial and complete ruptures).
3. Minimum of 16 years.

Participants eligible for this trial must **not** meet any of the following criteria:

1. Participant has a history of a previous ACL injury or knee surgery to the index knee.
2. Indication for acute surgery because of related injuries to the knee. (e.g. bucket handle meniscal tear that results in a locked knee or intra-articular fractures)
3. Female who is pregnant or plans to become pregnant in the first 4 months of the trial, since MRI assessment cannot be performed.

#### Study setting

This study will be performed in several Belgian hospitals: University Hospital of Leuven, Clinique Saint-Luc Bouge, Jessa Ziekenhuis Hasselt,, and University Hospital of Liège. Patients will be recruited at the Department of Orthopaedics and the Department of Physical Medicine and Rehabilitation of the participating sites. Additionally, in order to ensure the requested sample size, more sites will be selected in the course of the study.

#### Patient identification and screening

Patients introduced to any of the participating sites with an acute ACL injury will be assessed for eligibility. In habitual practice, patients who might have an ACL injury often present themselves at the emergency care unit and/or are referred to either (1) the Department of Physical Medicine and Rehabilitation or (2) the Orthopaedic Department. The screening and identification strategy will be performed similarly.

Like usual practice, during the initial consultation (V0), a physical examination of the knee including clinical tests to diagnose ACL injuries and a patient history will be taken by a medical doctor. Based on these findings the speculation of an ACL injury can be supported or rejected. If the clinical tests were positive, an MRI is scheduled to confirm the ACL tear. Additionally, if all other inclusion criteria are checked, the medical doctor will already inform the patient about the possibility to be included in the study.

During the second consultation (V1) with the medical doctor, the findings of the MRI are discussed. If the ACL tear is confirmed, the medical doctor asks if there is interest in participating in the study. If the patient wants to be involved in this study, he/she is referred to the appointed study assistant for further eligibility checks and extra information about the study. If the medical doctor confirms the eligibility and the patient agrees upon participation, informed consent will be signed by both the investigator and the patient.

To optimize recruitment and not miss potential candidates, the study nurse of the participating center will weekly check the planning of both the orthopedic and physical medicine department and remind the medical doctors of all potential candidates. Furthermore, regular contact with the emergency department is necessary to ensure that all relevant patients at the emergency department are correctly referred to either a consultation at the Department of Physical Medicine and Rehabilitation or the Department of Orthopedics.

Screening logs will be implemented at each recruiting site to document the reasons for non-inclusion in the study (e.g., the reason for decline or exclusion).

### Interventions

The study compares two standard treatment options for an acute ACL injury: [1] conservative treatment with optional delayed ACL reconstruction and [2] surgical treatment consisting of immediate ACL reconstruction and rehabilitation. We will not deviate from current practice, except for the randomization, to keep the trial pragmatic. Hence, the study does not predetermine the type of ACL reconstruction and patients will choose their own physiotherapist for the rehabilitation. However, to guarantee a minimum quality of rehabilitation, which is crucial for the integrity of the comparison, we will provide evidence-based guidelines and progression criteria for ACL rehabilitation to the physiotherapists.

#### Conservative treatment consisting of rehabilitation and optional delayed ACL reconstruction

##### Rehabilitation

Patients in this treatment arm will complete rehabilitation with their physiotherapist. As mentioned above, the physiotherapist will receive evidence-based guidelines and criteria for ACL rehabilitation. However, there is enough flexibility in how the physiotherapist wants to apply those guidelines in clinical practice. The guidelines are based on current literature [26–28]. The rehabilitation protocol involves three phases (see below), and progression is based on goal-based criteria, not time based. If the specific goals of the previous phase are achieved, patients can progress to the next phase. We ask the participant to fill in a modified version of the Exercise Adherence Rating Scale (EARS) [29] (see outcomes) to evaluate the quality of the rehabilitation, at every follow-up visit (visit 3–7, see Table 1). The EARS consists of several questions about different exercises performed, the intensity and frequency of the rehabilitation program, and barriers and facilitators for adherence to the predefined exercises.

**Table 1:** Overview of the rehabilitation guidelines per treatment arm. *The rehabilitation protocol consists of three phases. Each phase focuses on specific aims and progression criteria are provided to decide on progression to the next phase*

	Rehabilitation + optional delayed surgery	Immediate reconstruction + rehabilitation	Criteria for progression
**Phase 0**: Pre-operative phase	Not applicable	- Restore full knee extension - Activation hamstrings and quadriceps to avoid atrophy - Patient education - Instruction of post-operative exercises	
**Phase 1**: Acute phase	- Restore full knee extension, patella mobility, full flexion ROM - Eliminate effusion - Restore gait pattern - Improve muscle control and activation - Restore proprioception	- Restore full knee extension, patella mobility, start flexion ROM - Eliminate effusion - Restore gait pattern - Improve muscle control and activation - Restore proprioception	- Full passive knee extension, flexion: Active flexion ROM ≥ 115° - Minimal/ no joint effusion - Independent walking - Quadriceps strength 60% of contralateral side
**Phase 2**: Progressive strengthening + neuromuscular control	- Restore full knee ROM - Improve lower extremity muscular strength + endurance - Improve proprioception, balance and neuromuscular control	- Restore full knee ROM - Improve lower extremity muscular strength + endurance - Improve proprioception, balance and neuromuscular control	- Full ROM - Quadriceps strength 80% of contralateral side - Single leg hop test: 80% of contralateral side - No pain or effusion
**Phase 3**: Return to activity/sports	- Normalize lower extremity strength - Improve muscle power + endurance - Perform sport-specific drills - Gradually return to full sport	- Normalize lower extremity strength - Improve muscle power + endurance - Perform sport-specific drills - Gradually return to full sport	- Strength quadriceps + hamstrings > 90% contralateral side - Single leg hop test: >90% contralateral side

#### Indications for delayed surgery

If a patient of the conservative treatment group complains about consistent symptomatic instability of the knee that prevents rehabilitation progression, a delayed surgery can be considered. A positive pivot shift in combination with ACL insufficiency-induced instability and an additional MRI are needed to confirm the cause of instability (Criteria based on the KANON trial [1]). According to current practice, delayed surgery will not be performed within the first 12 weeks post-injury.

#### Immediate ACL reconstruction and rehabilitation

##### ACL reconstructive surgery

We will not impose any guidelines on the type of ACL reconstruction to keep the trial pragmatic. The decision on graft type and surgery technique is a clinical decision made by the orthopedic surgeon of the participating center. All surgery details will be noted in the patient register and can be retrieved if necessary. Although the study does not predetermine the type of surgery, we will impose strict criteria for the timing of the surgery. The immediate ACL reconstruction must be performed within 12 weeks after the ACL injury. This to avoid that patients of the immediate ACL reconstruction already had a considerable amount of pre-operative physiotherapy sessions, thus keeping a clear distinction between both groups.

##### Rehabilitation

The same goal-based rehabilitation protocol will be applied as in the intervention group (see Table 2). However, depending on the type of surgery, some time restrictions regarding range of motion and weight-bearing can be imposed by the surgeon. In that case, the physiotherapist has to slightly adapt the rehabilitation protocol. Rehabilitation starts the first days after surgery. Furthermore, also preoperative rehabilitation sessions can be performed.

**Table 2:** Overview trial procedures

Procedures/ Assessment	Screening	Randomisation + baseline assessment	Intervention	Follow-Up Visits				
Visits	V1	V2		V3	V4	V5	V6	V7
Timing (months)	< 8 weeks after injury	Baseline < 8 weeks after injury		4 months post-injury ± 14 days	7 months post-injury ± 14 days	12 months post-injury ± 14 days	24 months post-injury ± 14 days	36 months post-injury ± 14 days
Enrolment								
Eligibility screen	X							
Informed consent	X							
Randomisation		X						
Intervention								
1. Rehabilitation + optional delayed surgery			(X)^1^	(X)^1^	(X)^1^	(X)^1^	(X)^1^	(X)^1^
2. Immediate ACL reconstruction			X^2^					
Assessments								
MRI	(retrieved from patient record)			X				X
PROMS^#^		X		X	X	X	X	X
Adverse events				X	X	X	X	X
Isokinetic strength				X	X	X	X	X
Single leg hop for distance					X	X	X	X

Table 2: *Trial procedures for a single patient*

^*# l*^*the following patient reported outcome measures (PROMS) will be assessed at V2-V7: KOOS, return-to-sport, return-to-work, IPQ-R, TSK, EARS and quality of rehabilitation;*^1^Optional delayed surgery can occur after randomization; ^2^Immediate ACL reconstruction has to be performed within 12 weeks after injury

### Outcomes

#### Primary outcomes

The Knee Injury and Osteoarthritis Outcome Score (KOOS) QOL is the primary outcome of this study. The KOOS collects data on five knee-specific patient-centered outcomes: pain, symptoms, ADL, sport and recreational function (Sport/Rec), and knee-related QOL. Since most ACL patients report no or only small issues with pain, symptoms, and ADL function preoperatively there is less room for improvement on those subscales, and therefore the subscales Sport/Rec and QOL are recommended for follow-up ACL patients [30, 31]. We chose the subscale QOL as the primary outcome since not all participants will be involved in sports, and therefore the Sport/Rec is less useful in our trial. Standardized answer options are given on a Likert scale, and each question is assigned a score from 0 to 4. A normalized score is calculated for each subscale (100 indicating no symptoms and 0 indicating extreme symptoms). Test-retest reliability of the KOOS [32] is high with ICC values between 0.83 and 0.95 for KOOS QOL and between 0.61 and 0.95 for the other subscales. The minimal detectable change is 7-7.2 for the KOOS QOL and between 5 and 12 for the other subscales [32].

#### Secondary outcomes

##### The Knee Injury and Osteoarthritis Outcome Score (KOOS)

subscales: pain, symptoms, ADL, sport and recreational function [22].

##### International Knee Documentation Committee Subjective Knee Form (IKDC subjective)

this questionnaire administers patient’s perception of knee symptoms during activities of daily living and sports activities. This questionnaire consists of 18 items and a total score is calculated (range 0-100, with higher scores representing lower levels of symptoms, higher levels of function and participation) [33].

##### Modified Tegner Score

this scale will be used to grade the activity level of the patients [34]. A score is given from 1 (sick leave or disability pension because of knee problems) to 10 (competitive sports) based on the highest level of activities the patient performs regularly. Beside the current activity level, we will also ask the pre-injury activity level to determine when the patient returned to normal activities.

##### Illness perception questionnaire (IPQ-R)

the illness perceptions questionnaire measures patients’ beliefs and feelings about their illness (such as whether they think the illness can be cured or controlled by their treatment) [35].

##### Tampa Scale for Kinesiophobia

this questionnaire measures the fear of movement. It is a 11 item scale that uses a 4-point likert scale. Psychological factors such as fear of movement might be an important predictor of outcome after ACL injury, regardless of surgical treatment or not. Hartigan et al. (2013) showed for example that kinesiophobia is related to knee function after surgery [36].

##### The EQ-5D-5 L questionnaire

this questionnaire measures generic health status in 5 dimensions: mobility, self-care, usual activities, pain/discomfort and anxiety/depression. Each dimension has 3 levels: no problems, moderate problems and extreme problems. The sum of the score on the 5 dimensions describes the patient’s health state. We will administer this questionnaire for the economic evaluation to compute quality-adjusted life years (QALYs).

##### Functional tests

*The single leg hop for distance.* This functional test assesses knee functional performance. The patient will be asked to jump as far as possible on one leg (push-off and landing on same leg). After the landing, the patient has to keep balance for 3 s without shuffling on the stance leg. The patient will performs 3 trials on the injured leg and 3 trials on the uninjured leg. Subsequently the limb symmetry index (LSI) will be calculated by dividing the distance jumped on the injured leg by the distance jumped on the contralateral, uninjured leg [37]. This test is often used to decide on return-to-sport, with an LSI > 90% as criterium for readiness for return-to-sport [38].

*Isokinetic strength of the quadriceps and hamstrings*. Isokinetic strength of the quadriceps and hamstrings will be measured on an isokinetic dynamometer (Biodex or Cybex). This to assess recovery of strength. For both the quadriceps and hamstrings maximal concentric strength will be measured at 60°/sec and 240°/sec. Similar to the single leg hop for distance, the symmetry between the strength of the injured leg and uninjured leg will be calculated. Again an LSI of > 90% is often used as criterium for readiness for return-to-sport as a score < 90% is correlated with increased risk for re-injury risk after ACL reconstruction [38, 39].

##### Adverse events

Adverse events such as surgical complications, arthrofibrosis, infection and any additional acute injury to the ipsilateral or contralateral knee (such as re-injury, graft-rupture or contralateral ACL injury, laesions of menisci, cartilage or ligament,… ) will be registered at every follow-up visit.

##### Return to work/ return to pre-injury activity level

A customized questionnaire will be developed to administer the following data: days of sick-leave, previous and current occupational level, type of job, job-related activities, pre-injury sport level and time to return to pre-injury activity level.

##### Rehabilitation

A customized questionnaire is developed to collect information about the rehabilitation: the frequency (amount of sessions per week) and intensity of physiotherapy sessions, whether the rehabilitation involved active exercise therapy, the type of exercises and whether the patient performed exercises at home or in the gym. A list of exercises is provided. The patient has to check which exercises were performed during the rehabilitation. and whether the patient performed exercises at home or strength training (in a fitness center).

##### The anterior cruciate ligament OsteoArthritis score (ACLOAS)

This scoring system will be used to score and monitor structural knee joint damage on MRI [40]. The following joint features are assessed with this scoring system: acute osteo-chondral injuries, traumatic and degenerative bone marrow lesions, meniscus morphology and extrusion, osteophytes, collateral and cruciate ligaments including ACL graft, Hoffa-synovitis and effusion-synovitis. In addition to the MRI features assessed with the ACLOAS, the following MRI features will be assessed to detect recovery of the ACL: slope of ACL with respect to the Blumensaat line, distance between the Blumensaat line and the ACL, bounderies and tension of the ACL [16].

##### Central MRI reading

All pseudonymized MRI images will be scored by one central reader. This central reader will be a physician with expertise in musculoskeletal imaging. The central reader scores the images according to ACLOAS.

### Timeline

**Fig. 1:**
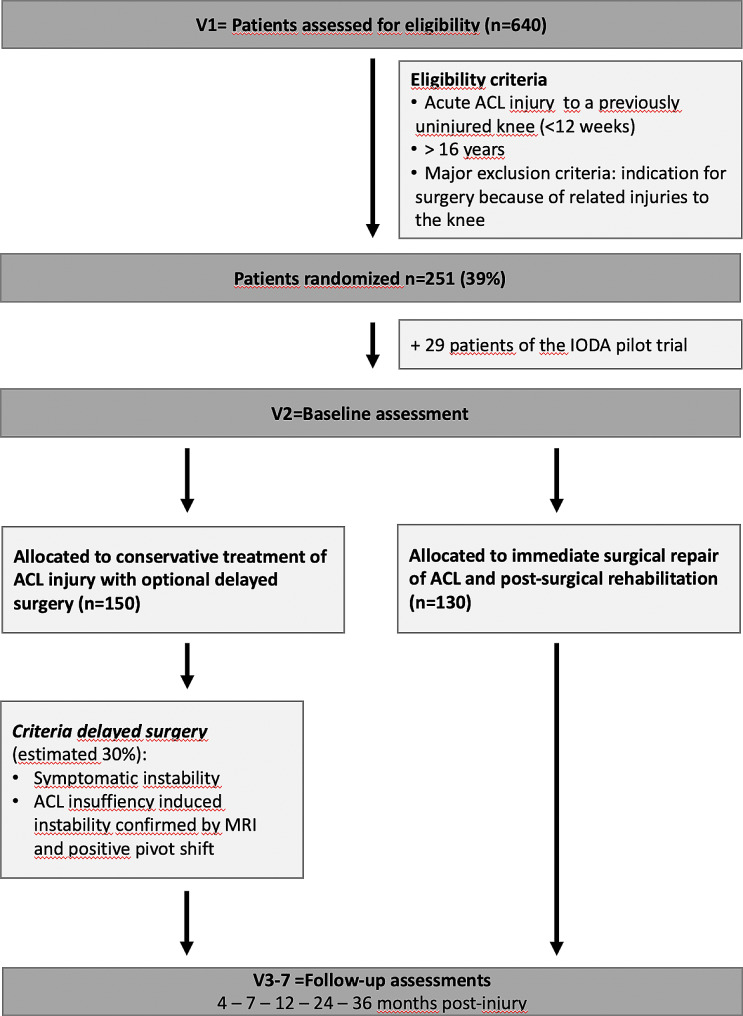
Trial flow chart V1 = visit 1

An overview of all assessments that will be performed at the different study visits can be found in Table 1. Five follow-up visits are planned: at 4 and 7, 12, 24 and 36 months after randomization see Fig. 1.

### Sample size

The outcome(s) on which the sample size calculation is based upon is the KOOS QOL, a self-administered questionnaire. The outcome for the **non-inferiority analysis** is the change on the KOOS QOL between the baseline measurement and 1 year after randomization. The sample size was calculated using the power procedure in SAS 9.4 to show with 80% of power that conservative treatment is not inferior to standard treatment, where a non-inferiority margin of 7 (change-points) was adopted based on the study of Collins et al. who report a minimal detectable change in KOOS QOL of 7 points [33]. The analysis consists in estimating the difference in mean change-scores between both treatment arms with a one-sided 95% confidence interval (Corresponding to a 5% significance level). Non-inferiority will be concluded when the lower limit of the confidence interval will be above the non-inferiority margin of -7. An equal treatment effect in both arms and a standard deviation for the change-score of 19 were assumed for the sample size calculation [1]. Assuming a drop-out of 10%, a total number of 260 patients is required for randomization. For the **superiority analysis** the sample size was calculated to demonstrate with a two-sample t-test a difference between the two treatment groups with respect to the change in KOOS QOL score at 7 months follow-up compared to post-injury. Considering equal group sizes, a two-sided 5% significance level, 90% power, the smallest important difference as 13.5 change-points [31] a standard deviation of 18 for the change in KOOS QOL versus baseline [31], and a 10% drop-out rate, the calculation indicates a total sample size of 88 patients. Furthermore, the 5% significance level was adopted since a hierarchy of statistical testing will be used. Only if the non-inferiority test is significant at 12 months, the test of superiority at 7 months will be performed since superiority is judged necessary to change practice. Furthermore, a prediction model will be built in the intervention group (rehabilitation and optional delayed ACL reconstruction) with the event of delayed surgery as binary outcome and where an initial set of the five predictor variables described above will be considered. Following the rule of thumb of including at least 10 events per parameter in the model, this analysis requires at least 50 events (i.e. delayed surgeries in the experimental group). Assuming that 1/3 of patients in the experimental group will need delayed surgery (KANON Trial), a minimum of 150 patients will be needed in the intervention group in order to develop such a prediction model.

### Randomization

Random sequence generation (computer generated, using block randomisation) will be performed and allocation will be concealed. An allocation ratio of 1:1.15 will be applied (130 patients allocated to immediate ACL reconstruction and 150 patients allocated to conservative treatment). With this we comply with the sample size calculation. We will have (1) minimum 130 patients per arm for the primary analysis and (2) 150 patients in the conservative arm for the secondary analysis. At each participating site, the responsible study nurse/principal investigator will have access to the randomisation tool in REDCap.

To ensure the integrity of the Trial: the randomization list will be prepared by a statistician not involved in the trial. The randomization list will be incorporated in the data management tool ‘REDCap’. After all patients have finished the Trial and the database is locked, the randomization code will be broken for analysis of response data.

### Blinding

Given the nature of the trial, blinding of participants and care providers is not feasible.

Data collectors and data analysts will be blinded to the extent possible. Outcomes will be collected in the same way in both groups, e.g. by electronical questionnaires for which assessors and collectors can be blinded. However, because of the subjective and self-reported nature of the outcomes being assessed, detection bias may be a potential risk of bias. After all participants have completed the Trial, the database will be locked and the collected Trial data will be unblinded to allow analysis of the Trial data.

### Statistical analyses

The primary analyses will be performed on the intention-to-treat (ITT) population (subjects are included in the groups to which they were randomly assigned, even if they did not complete their treatment). For example, subjects in the conservative group that underwent ACL reconstruction or subjects in the early reconstruction group that did not undergo reconstruction. This choice was based on the main aim of this trial: informing medical doctors which strategy (immediate ACL reconstruction or conservative with optional delayed surgery) is most appropriate after acute ACL injury and therefore help them in decision-making to undergo immediate ACL reconstruction or not. This ITT analysis will closely reflect everyday practice as in real life; patients will not always adhere to either therapy’s advice.

The as-treated population includes subjects in groups as to how they were treated. This post- hoc as-treated analysis will result in 3 groups: rehabilitation alone, rehabilitation plus early ACL reconstruction, and rehabilitation plus delayed ACL reconstruction. However, this analysis will only be explorative as this will be a non-randomized comparison that will probably be biased.

#### Primary outcome analysis

The primary analysis will compare the intervention group with the control group on their mean change in KOOS QOL at 2 primary endpoints:

1. Non-inferiority testing at 1 year post-injury.
2. Superiority testing at 7 months post-injury.

A one-sided 95% confidence interval for the mean difference between groups excluding 7 units in the disadvantage of the conservative treatment will be considered as a demonstration of non-inferiority at 1 year. A two-sided 95% confidence interval for the mean difference between groups excluding 13.5 units in the advantage of the conservative treatment will be considered as a demonstration of superiority at 7 months.

Estimation and testing of these mean differences will be based on a single analysis model. A linear model for repeated measurements will be used, applied to outcome measures at all time points, modelling an unstructured residual variance-covariance matrix to deal with correlations due to the longitudinal data structure. Compared to separate cross-sectional analyses, this approach improves the power and minimizes the effects of possible bias due to drop-out. All patients with at least one outcome measurement are included in the analysis, even if they had no outcome measured at 7 or 12 months. The model includes group, time-point and group by time-point interaction as effects. The mean differences between the groups at 7 and 12 months and their confidence intervals will be estimated as post-hoc tests based on this model. Given the hierarchical approach of statistical testing (superiority testing only if non-inferiority is demonstrated), no correction for multiple testing will be performed.

The primary analysis will be applied to the ITT population. Patients without outcome measures apart from baseline will be excluded.

#### Secondary outcome analysis

Mean differences at other time-points are obtained as additional post-hoc tests from the model used for the primary analysis. The analysis of secondary outcome variables will be performed analogously to the primary analysis. A sensitivity analysis will be performed, repeating the primary analysis using multiple imputation to deal with patients that have no outcome measures apart from baseline.

#### Prediction analysis

A prediction model will be ran on the intervention group (rehabilitation with optional delayed ACL reconstruction). Based on 5 variables we want to assess whether it is possible to predict whether the patients in the intervention group will need surgery or not (binary outcome). The event of ‘delayed surgery’ was considered as binary outcome and not as time-to-event since the main question is to investigate whether it is possible to predict who will need a delayed surgery. The timing of the delayed surgery is less relevant as the timing is often influenced by several personal factors such as work, school, holiday and also by the availability of the surgeon etc.

A logistic regression model will be used and a backward selection procedure will be applied for model selection. The performance of the model will be quantified by the area under the ROC curve (AUC). Internal validation will be performed by means of a leave-one-out cross validation method to obtain a more realistic estimate of the model performance.

#### Explorative analysis

A similar method of analysis as described for the primary analysis (intention-to-treat) will be applied to the as-treated analyses.

### Data monitoring

#### Access to data

The investigator will permit trial-related monitoring, audits, ethics committee review, and regulatory inspection, providing direct access to all related source data/documents.

At the end of the trial, the funder (KCE) will have access to the study data. This will only be the pseudonymized study data.

### Safety recording and reporting

The risk of adverse events (AE) occurring due to the intervention in this trial is unlikely. Therefore, safety reporting will be limited to the safety reporting necessary for routine care. The participant will be asked to report any adverse event related to the study-specific intervention to the study team. The following adverse events will be registered at every follow-up visit: surgical complications, arthrofibrosis, infection, any additional acute injury to the ipsilateral or contralateral knee (such as re-injury, graft-rupture or contralateral ACL injury, lesions of menisci, cartilage or ligament,… ).

These reported events will be documented by the investigator in the source documents. In addition, the following minimum information should be recorded for each adverse reaction by the reporting investigator (AE description, start and stop date of the AE, severity, seriousness, causality assessment to the study interventions, and outcome). The sponsor will keep detailed records of all AEs reported to him by the investigators and will evaluate for seriousness, causality, and expectedness.

### Protocol amendments

Any modifications to the protocol which may impact the conduct of the study, the potential benefit of the patient, or may affect patient safety, including changes in study objectives, study design, patient population, sample sizes, study procedures, or significant administrative aspects will require a formal amendment to the protocol. The trial steering committee will agree upon such amendment, add to the trial registration on clinical trials.gov, and approve by the Ethics Committees before implementation.

## Discussion

Although two earlier RCTs (KANON [1] and COMPARE trial [2]) showed that conservative treatment with optional delayed surgery does not result in inferior clinical outcomes compared to immediate ACL reconstruction, most ACL patients in many countries still undergo immediate ACL reconstruction.

This lack of translation of research findings to clinical practice might be attributed to several reasons. One important reason is that evidence is still scarce as only 2 RCTs are performed (total of 288 patients were included). Furthermore, the fact that there are no evidence-based decision criteria that helps clinicians to decide on treatment choice, might let patients and physicians choose for surgery, to avoid potential conservative treatment failure and the associated prolonged rehabilitation.

Therefore, the first aim of this additional RCT, is to verify the results of the two previous RCTs in a large and pragmatic setting. We will aim to include 280 ACL patients, this sample size is twice as large as the population investigated in the previous RCTs. Second, this RCT will be the first adequately powered study to investigate whether patient-specific factors could predict which patients benefit from conservative treatment and which patients benefit from immediate ACL reconstruction. Early patient identification seems very important to further improve ACL treatment. In the first place, early classification will improve clinical outcomes and allow patients to return to work/sport more quickly. The patients who require surgery undergo timely surgery, and in this way, a double rehabilitation period is avoided. In addition, patients who are good candidates for non-surgical treatment do not undergo unnecessary surgery. There will be no additional iatrogenic trauma due to surgery, resulting in a faster return to pre-injury activities. Identifying patients early on also has the added benefit of reducing costs, as the most cost-effective strategy is to provide patients with optimal treatment as soon as possible. Eggerding et al. showed that rehabilitation and delayed surgery have the highest cost and potentially the longest trajectory to restore full function and should thus be avoided [41].

To develop patient identification guidelines, one should use prognostic risk modeling. Such models can aid clinicians in making treatment decisions based on their clinical profile. So far, such evidence does not yet exist. In the IODA trial, we will try to predict delayed ACL reconstruction based on factors such as patient symptoms, pre-injury activity level, MRI features, and patients’ beliefs. Our ultimate aim is to provide outcome probabilities for different combinations of predictors. This will enable care providers to estimate the risk of a patient failing non-operative treatment. Based on this information, a well-concerned, evidence-based, shared decision can be made. This is in contrast with current practice, where clinicians still rely on their gut feeling, clinical experience, and patient’s beliefs in choosing an appropriate treatment.

### Trial status

Prior to this study, we conducted a pilot study to investigate recruitment feasibility. In this pilot study we already recruited 29 patients. These patients will also be proposed to participate in the full trial for further follow-up. Recruitment for the IODA study started in March 2023 and is ongoing at the time of manuscript submission. Recruitment is expected to be completed by April 2025.

### Electronic supplementary material

Below is the link to the electronic supplementary material.

**Additional file 1**: SPIRIT 2013 Checklist: Recommended items to address in a clinical trial protocol and related documents

## Data Availability

Data sharing is not applicable to this article as data collection is still ongoing.

## Abbreviations

ACL: Anterior cruciate ligament
RCT: Randomized controlled trial
MRI: Magnetic resonance imaging
KOOS: Knee injury and osteoarthritis outcome score
QOL: Quality of life
EARS: Exercise adherence rating scale
ROM: Range of motion
PROMS: Patient-reported outcome measures
ADL: Activities of daily living
SLHD: Single leg hop for distance
RTS: Return to sport
ACLOAS: Anterior cruciate ligament osteoarthritis score
TSK: Tampa scale of kinesiophobia
IPQ-R: Illness perceptions questionnaire (revised)
AE: Adverse events
GCP: Good clinical practice
KCE: Belgian Health Care Knowledge Centre
GDPR: General data protection regulation

## Declarations

### Status of the study

Patient recruitment is ongoing in the University Hospital of Leuven, CHU Liège, Jessa Ziekenhuis, and Clinique Saint Luc Bouge 35 patients are randomized (29 pilot study + 6 full study).

### Confidentiality

Personal data recorded on all documents will be regarded as confidential. All data will be handled and stored in accordance with the General Data Protection Regulation (GDPR) and the Data Protection Act 2018. During the clinical trial and after trial closure, adequate and accurate records will be maintained to enable the conduct of a clinical trial and the quality of the research data to be evaluated and verified. All essential documents will be stored to ensure that they are readily available, upon request, for the minimum period required by national legislation or for longer if needed.
